# Telemedicine Strategy to Rescue CPAP Therapy in Sleep Apnea Patients with Low Treatment Adherence: A Pilot Study

**DOI:** 10.3390/jcm10184123

**Published:** 2021-09-13

**Authors:** Onintza Garmendia, Ramon Farré, Concepción Ruiz, Monique Suarez-Girón, Marta Torres, Raisa Cebrian, Laura Saura, Carmen Monasterio, Miguel A. Negrín, Josep M. Montserrat

**Affiliations:** 1 Sleep Unit, Hospital Clínic-Universitat de Barcelona, 08036 Barcelona, Spain; onintzag@gmail.com (O.G.); csanchez@clinic.cat (C.R.); mcsuarezgiron@gmail.com (M.S.-G.); 2 Unitat de Biofisica i Bioenginyeria, Facultat de Medicina i Ciencies de la Salut, Universitat de Barcelona, 08036 Barcelona, Spain; rfarre@ub.edu; 3 CIBER de Enfermedades Respiratorias, 28029 Madrid, Spain; torreslopezmarta@gmail.com; 4 Institut Investigacions Biomediques August Pi Sunyer, 08036 Barcelona, Spain; 5 Agency for Health Quality and Assessment of Catalonia (AQuAS), 08005 Barcelona, Spain; 6 CIBER de Epidemiología y Salud Pública, 28029 Madrid, Spain; 7 Esteve Teijin, 08029 Barcelona, Spain; rcebrian@esteveteijin.com (R.C.); lsaura@esteveteijin.com (L.S.); 8 Multidisciplinary Sleep Unit, Department of Respiratory Medicine, Hospital Universitari de Bellvitge, 08907 L’Hospitalet de Llobregat, Spain; cmonasterio@bellvitgehospital.cat; 9 Quantitative Methods Department, TiDES Institute, Las Palmas de Gran Canaria University, 35001 Las Palmas de Gran Canaria, Spain; miguel.negrin@ulpgc.es

**Keywords:** obstructive sleep apnea, sleep breathing disorders, nasal pressure, patient adherence, compliance, telemedicine interventions

## Abstract

Patients with sleep apnea are usually treated with continuous positive airway pressure (CPAP). This therapy is very effective if the patient′s adherence is satisfactory. However, although CPAP adherence is usually acceptable during the first months of therapy, it progressively decreases, with a considerable number of patients accepting average treatment duration below the effectiveness threshold (4 h/night). Herein, our aim was to describe and evaluate a novel telemedicine strategy for rescuing CPAP treatment in patients with low adherence after several months/years of treatment. This two-week intervention includes (1) patient support using a smartphone application, phone and voice recorder messages to be answered by a nurse, and (2) daily transmission and analysis of signals from the CPAP device and potential variation of nasal pressure if required. On average, at the end of the intervention, median CPAP adherence considerably increased by 2.17 h/night (from 3.07 to 5.24 h/night). Interestingly, the procedure was able to markedly rescue CPAP adherence: the number of patients with poor adherence (<4 h/night) was considerably reduced from 38 to 7. After one month, adherence improvement was maintained (median 5.09 h/night), and only 13 patients had poor adherence (<4 h/night). This telemedicine intervention (103€ per included patient) is a cost-effective tool for substantially increasing the number of patients with CPAP adherence above the minimum threshold for achieving positive therapeutic effects.

## 1. Introduction

The obstructive sleep apnea (OSA) syndrome is a highly prevalent chronic disorder associated with substantial morbidity, resulting in considerable healthcare costs [1,2,3]. Continuous positive airway pressure (CPAP) is by far the most widespread and effective therapy for OSA and is thus the gold standard treatment for this sleep breathing disorder [4]. However, suboptimal patient adherence to CPAP is common [5,6] despite using conventional interventions to increase it [7]. Regarding the clinical effectiveness of CPAP, it is important to mention that treatment adherence of 4 h per night is currently considered the minimum required. However, data in the literature describing the dose-response relationship between CPAP usage and improved clinical outcomes (including sleepiness, functional status, and hypertension) strongly suggest setting an adherence threshold of >5 h/night [8,9,10]. Therefore, it is of crucial importance to increase CPAP adherence as much as possible to achieve optimal treatment effectiveness.

Telemedicine is a strategic approach to address public health challenges in chronic diseases, offering potential cost-effective management options [11]. In the case of sleep medicine, and particularly in OSA, multiple telemedicine modalities can be used, including telediagnosis, teleconsultation, and telemonitoring of patients being treated with CPAP. However, it is crucial to carefully select clinical outcomes and adequately target those patients who may benefit from telemedicine interventions [12,13,14]. Currently, and especially after experiencing a global pandemic with COVID-19, the use of telemedicine has been markedly increased. Telemetric monitoring of OSA patients allows remote CPAP titration [14,15]. Moreover, telemedicine allows that most patients can be remotely contacted (by phone or video-visits) for satisfactorily managing OSA [14,16]. Recently described telemedicine interventions focused on improving CPAP adherence are applied during the first weeks/months after CPAP is prescribed, when adherence is relatively acceptable [15,16]. However, it is well known that the patient’s adherence with this treatment decreases [16,17,18,19,20,21,22] over time. Therefore, novel interventions addressed to improve CPAP adherence in patients already in long-term treatment are required.

Hence, the aim of this research was to set a specific telemedicine procedure for rescuing CPAP adherence in patients already on treatment who, regardless of being conventionally followed up by hospital or CPAP provider staff, are poorly compliant as indicated by a low number of hours per night on CPAP. The primary end-point was to increase the percentage of rescued patients presenting at baseline treatment adherence lower than 4 h/night (considered poor). The secondary end-point was to also improve the percentage of patients who achieve optimal adherence among those with acceptable adherence (4–5.5 h/night).

## 2. Materials and Methods

### 2.1. Patients

This was a prospective, pre-post intervention, single-arm study that evaluated patients (18–75 years old) that had a CPAP prescription from September 2016 to June 2020. The research and analysis of the telemedicine application was performed from November 2020 to April 2021 on patients who did not comply with either a minimum (<4 h) or suboptimal (4–5.5 h/night) CPAP treatment despite careful follow-up by the hospital and the service provider. To this end, the value of CPAP adherence registered in the home CPAP device used by the patient was considered.

Before entering the study, the patients were followed up according to our usual protocol. Briefly, before starting CPAP treatment, patients participated in a 1.5-h educational and training session (theoretical and practical use of CPAP and selection of an adequate mask). After starting with CPAP treatment, the first visit was at 15–30 days, the second one after 3 months, the third visit was after 6 months, and finally a fourth visit 1 year after prescription. If treatment was satisfactory, the patient was visited alternately by the specialized nurse and the provider each year. Patients attended their regular medical visits depending on medical needs and problems about CPAP. According to nurse or physician criteria, patients with inadequate adherence were visited (individually or in a group), usually every 3 months. If required, the CPAP provider company increased the number of patient visits.

Patients who met the inclusion criteria and signed the informed consent were included. The exclusion criteria were severe associated comorbidities or coexisting severe psychiatric disease, central apneas, pregnancy, regular use of sedatives or narcotics, uvulopalatopharyngoplasty, incapacity to carry out questionnaires, and any contraindication for CPAP therapy. Importantly, low experience in the use of smartphones or internet applications was not an exclusion criterion. Thus, only patients with no previous experience in using these communication tools were excluded.

### 2.2. Intervention

The intervention was based on the following three components: (1) Each patient received an automatic-CPAP device (Dreamstation, Respironics) which was able to remotely transmit data on CPAP pressure, breathing flow, air leaks, treatment adherence, and residual respiratory events to a commercially available web server providing remote monitoring to the health care provider. The setting also allowed remotely changing the value of CPAP pressure applied, thus performing home accurate titration/retitration if required [15]. The patient was asked to use a specially designed smartphone application (APPnea) [12] to promote patient self-monitoring of CPAP treatment. APPnea asked the patient simple questions on adherence, sleep improvement, CPAP side effects, and general lifestyle perception each other day. This questionnaire is provided as a Supplementary File Table S1. All answers were sent to a web server and evaluated by a specialized nurse who contacted the patient if required [12]. The patient was invited to use a voicemail available 24 h to collect the patient’s questions or problems. Patients were encouraged to leave voicemail messages to be checked and eventually answered by a specialized nurse.

The nurse communicated with the patient if data transmitted by the CPAP device showed problems (air leaks, high residual events, or poor adherence). The telemedicine intervention using the described procedure lasted 15 days. CPAP adherence was measured immediately after the intervention and after a 30-day subsequent period. Moreover, the costs of the intervention as well as the patient’s satisfaction were assessed.

### 2.3. Data Analysis

A per-protocol analysis of improvement in CPAP adherence after the intervention (pre-post analysis) was carried out. Data were characterized by mean (SD) for continuous variables with normal distribution, median (Q1; Q3) for those with nonnormal distribution, and number and percentage of patients for categorical variables. Ninety-five percent confidence intervals for overall incidence in adherence rate and mean change from baseline in adherence measured in hours were computed. Paired adherence data before the intervention (PRE), after the intervention (POST), and 1 month after the end of intervention (1-MONTH) was analyzed with nonparametric ANOVA using the Friedman test followed by Dunn’s multiple comparisons test (Prism, GraphPad, CA, USA). If the Friedman test was significant, post hoc paired comparison was made using the Wilcoxon signed-rank test. The McNemar–Bowker test was used to determine differences on a categorical variable between two related groups. All tests were two-tailed, and significance was set at 0.05. All analyses were performed with IBM SPSS Statistics version 26.0 (Armonk, New York, NY, USA).

### 2.4. Cost Analysis

The cost of the different steps followed in the intervention were considered: before the start of the intervention, a total cost of 3000 € corresponding to the APPnea application was distributed among the 56 patients. Change to an automatic-CPAP was also necessary for 17 patients. Both items resulted in a baseline cost of 4330.25 € (3000 + 78.25 × 17), 77 € per-protocol patient. The other costs include the remote monitoring time (in minutes) by a specialized nurse; the cost of the first phone visit (in minutes), and the other contacts with a specialized nurse through voice mail messages, emails (assumed 5 min per message) and phone calls (in minutes); and time (in minutes) of the two visits of the service company providing CPAP equipment plus additional visits for mask replacements. Unit costs were the same used in similar recent studies [11,15]: 16.2 €/hour for a specialized nurse, 19 €/hour for a technician of the provider company, and 24 € for mask replacement.

## 3. Results

**Figure 1:**
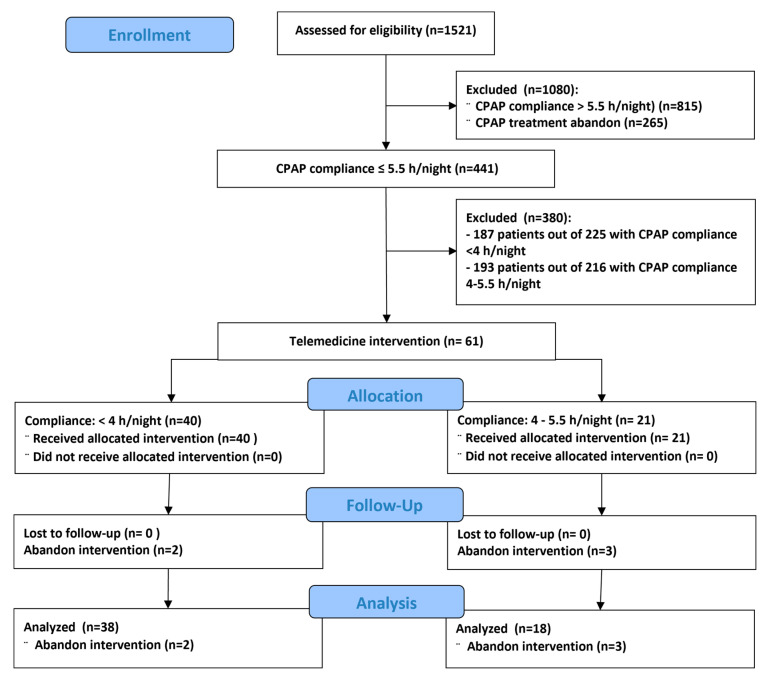
Flow chart of the study.

Table 1 shows the patient’s characteristics and their mild comorbidities (patients with severe comorbidities were excluded). Sixty-one patients participated in the study, of which five dropped out from the protocol because they voluntarily abandoned the telemedicine procedure, thus 56 patients completed the study as indicated by the flow chart in Figure 1. Only 7% of patients required CPAP retitration and, in that case, nasal pressure was remotely adjusted, 23% of patients used the nurse call line and only one patient needed a new mask.

**Figure 2:**
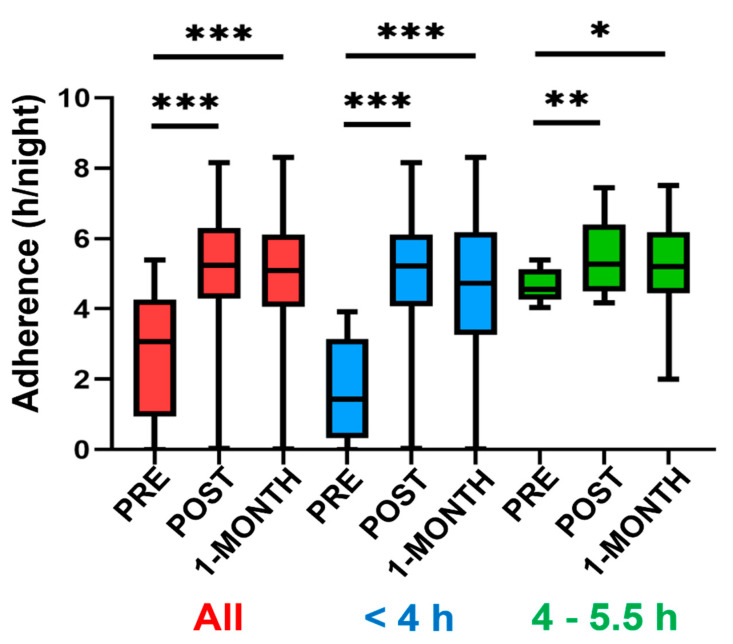
Adherence of CPAP is expressed as the number of hours per night on treatment (median, 25–75% percentiles and smallest and largest values). Data are shown for the whole group of patients (red), for those patients with preintervention (PRE) adherence of <4 h/night (blue), and for those with preintervention adherence between 4 and 5.5 h/night (green). Labels “POST” and “1-MONTH” indicate values measured immediately at the end of the intervention and 1 month later, respectively. All changes from PRE to POST and from PRE to 1-MONTH were statistically significant, and none of the minor changes from POST to 1-MONTH were statistically significant. ***, **, and * indicate *p* < 0.001, *p* < 0.01 and *p* < 0.05, respectively.

Figure 2 shows the CPAP therapy adherence quantified as the number of hours per night. By considering all patients, on average, the telemedicine intervention considerably increased median treatment adherence by 2.17 h/night (from 3.07 to 5.24 h/night). Among the group of patients with baseline adherence < 4 h/night, the median increase was even higher: 3.79 h/night (from 1.44 to 5.23 h/night). Even patients with already acceptable adherence (4–5.5 h/nigh) experienced an increase of 0.72 h/night (from 4.55 to 5.27 h/night). All these changes were statistically significant. Most interestingly, the general pattern of increase in adherence observed just at the end of the intervention did not significantly change after 1 month: median adherences were 4.55, 5.27, and 5.21 h/night in the three groups respectively (Figure 2). Full raw data on CPAP adherence are provided in a Supplementary File Table S2.

The marked increase in adherence (Figure 2) resulted in a considerable number of patients with rescued CPAP treatment.

**Figure 3:**
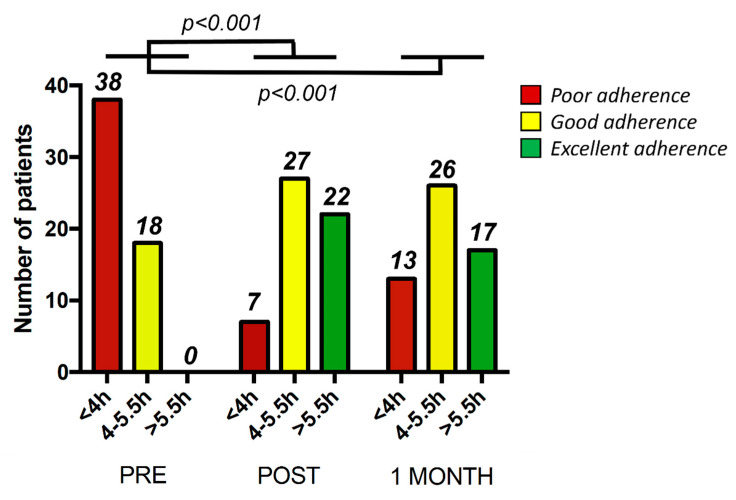
Number of patients within three different levels of adherence: poor and thus poor adherence (<4 h/night) (red), good adherence (4–5.5 h/night) (yellow) and excellent adherence (>5.5 h/night) (green) at three different time points: prior to the telemedicine intervention (Pre), after the intervention (Post) and one month later (1 month). The *p* values refer to differences in the number of patients within three different levels of adherence when comparing PRE vs. POST and PRE vs. 1-MONTH.

Figure 3 shows, for each time (preintervention, postintervention and 1 month later) what was the number of patients exhibiting three different levels of adherence: poor adherence (<4 h/night), good adherence (4–5.5 h/night) and excellent adherence (>5.5 h/night). Remarkably, the most important result is that the number of patients with poor adherence (<4 h/night) was considerably reduced from 38 to 7 and 13 after the intervention and after a subsequent month, respectively. In addition to markedly reducing the number of patients with poor adherence, the intervention increased the number of patients with good adherence (4–5.5 h/night) from 18 to 27 and 26, respectively. Finally, whereas before the intervention no patient had optimal adherence (>5.5 h/night), after the intervention and 1 month later the number increased to 22 and 17, respectively. The number of patients within three different levels of adherence when comparing PRE vs. POST and PRE vs. 1-MONTH was significantly different (*p* < 0.001 in both cases; Figure 3).

**Figure 4:**
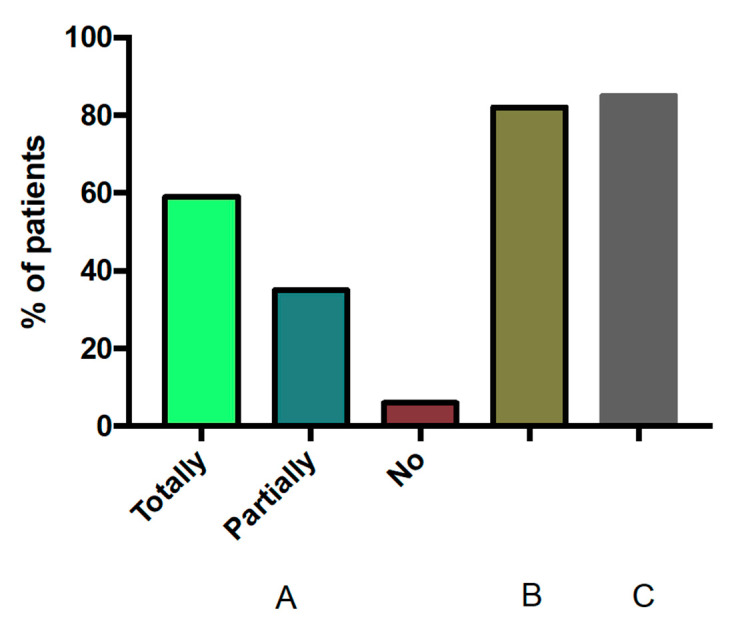
Patient satisfaction with the telemedicine intervention. Left section (**A**) shows the percentage of patient’s responses when asked whether the App was totally, partially or not useful. (**B**) and (**C**), on the right, show the percentage of patients who would recommend using the App to other patients and who would like to use the App regularly along their CPAP treatment, respectively.

Patients showed considerable satisfaction with the intervention: they answered that the questions periodically posed to them by the smartphone App were partially (39%) or totally (60%) useful, with only one patient answering negatively (Figure 4). Interestingly, 82% of patients would recommend the application to other patients and, 85% would like to regularly use the application to control their CPAP therapy (Figure 4).

**Figure 5:**
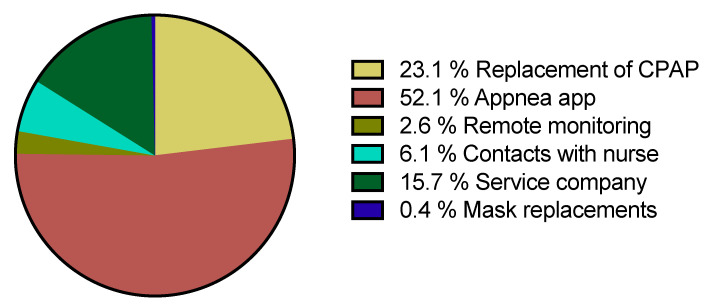
Cost distribution of the proposed telemedicine intervention.

The total cost of the intervention was on, average 103 €, per included patient. Figure 5 shows the cost distribution among the different intervention actions. No differences in individual costs were observed between patients with and without rescued CPAP adherence.

## 4. Discussion

In this study, we report that noncompliant CPAP treatment in patients with OSA who are under long-term therapy (average 2.5 yr) can be markedly rescued by means of a two-week telemedicine intervention which is based on two different points. First, personal support by using a smartphone application [12], phone and voice recorder where the patients could leave messages that were answered by a nurse. Second, CPAP device signal transmission from the patient’s home (e.g., pressure, residual events, adherence, air leaks) which allows remotely adjusting the nasal pressure applied if required. As shown in Figure 2 and Figure 3, the intervention was able to considerably increase the time that patients were on CPAP and thereby improving the level of adherence, in many cases above the threshold for therapeutic effectiveness (4 h/night).

Several previous publications have raised the clinical problem of poor adherence of CPAP in OSA. Pepin et al. [22] analyzed the CPAP therapy of OSA patients from a French nationwide database analysis (n: 480,000 subjects) and found that overall CPAP termination rates after 1, 2 and 3 years were 23.1, 37.1 and 47.7% respectively and raised the importance of phenotyping and personalized care approaches that determine the most appropriate. In the SAVE study, McEvoy et al. [6] found that CPAP adherence at the beginning of the study was 5.3 h/night, and after 24 months of follow-up, it was reduced to 3.4 h/night (n:1121, 5 different countries). In a similar study, Peker et al. [23] described that cardiovascular improvement was found only in subjects with good adherence. Bakker et al. [24] raised two important points: how many hours of CPAP use per night are necessary to improve symptoms and to reduce cardiovascular risk, and what strategies could be implemented to optimize adherence in clinical settings. The main conclusions were that combining theory-driven behavioral approaches with telemedicine technology could hold the answer to increasing real-world CPAP adherence rates, although randomized studies are still required, and socioeconomic barriers to telemedicine will need to be addressed to promote health equity. Accordingly, there is ample consensus on the need to improve the adherence of CPAP treatment to levels higher than the commonly observed in clinical practice.

Given the importance of the problem of poor CPAP adherence, different telemedicine interventions have been proposed to diminish or solve it. Aardoom et al. performed a meta-analysis [21] designed to investigate the effectiveness of a broad range of eHealth interventions in improving CPAP treatment adherence. The main conclusions were that eHealth interventions for adults with OSA could improve adherence to CPAP at the initial weeks/months after the start of treatment, increasing the mean nightly duration of usage by about half an hour. Uncertainty still exists regarding the timing, duration, intensity, and specific types of health interventions that could be most effectively implemented by health care providers [18]. In a recent randomized study, the effect of telemedicine applied after 3 months of regular treatment was analyzed. After 6 months of follow-up, the telemedicine group improved adherence [25]. However, to the best of our knowledge, data in the literature do not describe any procedure that, similar to the one presented herein, successfully rescues CPAP therapy adherence in patients with no recent prescription of CPAP but on therapy for a long period.

In the performed cost analysis, we estimated 103 € per-protocol patient, resulting in a cost per recovered patient of 152 €. It is noteworthy that these costs are overestimated since we divided the total cost of the smartphone application used in the intervention (APPnea) (3000 €) among the only 56 patients of the study. If this intervention was implemented for much more patients, the distributed cost of the App (which in this study was the most expensive contribution in total cost, as shown in Figure 5), would become negligible and then would virtually disappear. Therefore, the effective cost of the intervention per patient would be considerably reduced. To more precisely evaluate whether this intervention is cost-effective, it would be required to also consider that the associated increase in patient’s health represents fewer costs to the society, which seems to be proven in the literature. Indeed, Rossi et al. [26] showed that untreated OSA used more medical services and more medicines. Specifically, Guest et al. [27] and Kapur et al. [28] estimated that untreated OSA leads to a twofold increase in medical expenses in Europe and the USA, and Knauert et al. [29] obtained a similar conclusion by reviewing the topic.

The current study has limitations. One of them is that, although being multicentric, the number of patients is relatively reduced. The reason is that patients who were acceptable according to the inclusion criteria were very difficult to recruit since the protocol was carried out during the COVID-19 pandemic. This fact explains why 380 patients were excluded (Figure 1). However, under these conditions the telemedicine approach was tested in a realistic scenario characterized by severely reduced possibility of in person interaction between patients and health care staff. Another limitation is that the time of the follow-up after the intervention (one month) was reduced. However, in this pilot study we have demonstrated that the intervention is feasible and useful, and as such it can be easily reproduced to rescue CPAP treatment in patients poorly complying with the treatment. Regardless of the specific limitations of this study, it should be considered that telemedicine per se has limitations and cannot be applied without previously considering its potential drawbacks and requirements, specifically in the field of CPAP for OSA patients [30]. For instance, the requirement of training the health care professionals involved, the need of phenotyping which patients should be included in a telemedicine program, or better defining not only the cost for the health system but the social and labor costs saved by correctly treated patients.

## 5. Conclusions

To our knowledge, this is the first study that deals with the CPAP adherence rescue concept in patients under long-term treatment. The results obtained demonstrate that it is possible that a significant proportion of patients with poor, and thus inefficient, adherence achieve the minimum threshold of 4 h/night on CPAP. Moreover, patients already on an adherence range which was satisfactory but not optimal (4–5.5 h/night) increased adherence to optimal values (>5.5 h/night). The fact that the procedure is cost-effective and the very positive patient satisfaction strongly suggests that the proposed telemedicine intervention will be a powerful tool for improving CPAP usage in the clinical arena of OSA treatment.

## Figures and Tables

**Table 1:** Patient characteristics.

Number	56
Gender (male; %)	78.6
Age (yr; m ± SD)	57.9 ± 8.9
Apnea-hypopnea index (events/h; m ± SD)	45.8 ± 20.1
Time on CPAP therapy (yr; m ± SD)	2.46 ± 0.90
Main Comorbidity:	
Cardiovascular (%)	41.1
Metabolic (%)	39.3
Neurological (%)	1.8
Respiratory (%)	19.6
Depression (%)	12.5
Neurological (%)	1.8

## Data Availability

The details of data presented in this study are available on request from the corresponding author.

## Supplementary Materials

The following are available online at https://www.mdpi.com/article/10.3390/jcm10184123/s1, Table S1: Follow up patient questionnaire with 9 single-choice questions (Yes/No) concerning: (a) CPAP use and effectiveness (1–3), (b) common side effects (4–6), c) exercise, diet (7–9) and a final question to write down current weight (10). Table S2: CPAP compliance (h/night).
