# Supplementary material for: Telemedicine Strategy to Rescue CPAP Therapy in Sleep Apnea Patients with Low Treatment Adherence: A Pilot Study
Source: J Clin Med. 2021 Sep 13;10(18):4123. doi: 10.3390/jcm10184123 (PMC8470548; doi:10.3390/jcm10184123)
Supplement: Supplementary file 1 [file jcm-10-04123-s001.zip › Questionaire.pdf]

Supplementary tables

Table S1. Follow up patient questionnaire with 9 single-choice questions (Yes/ No) concerning: a) CPAP use and effectiveness (1-3), b) common side effects (4-6), c) exercise, diet (7-9) and a final question to write down current weight (10).

Questions	Automated answers
1) Have you used CPAP at least 4 hours every night?	Answer: No<br>Sometimes it takes a few weeks to adapt. Some patients put on their CPAP for a while during the day to get used to it. Do not obsess and try to be patient.<br><br>Answer: Yes<br>Fantastic! The more hours you use CPAP, the better.<br><br>*The information provided by Appnea-Q does not replace a medical opinion. If you still have doubts or problems persist after a week contact us through voicemail at xxxxx or send an email to xxx@xxx.cat to assess if you need a face-to-face visit. (Repeats in all recommendation messages)*
2) Have your general condition improved with CPAP treatment?	Answer: No<br>Do you use the CPAP enough hours? You must use it at least 4 hours, although the optimum is 6 hours. If you do not notice improvement despite wearing the CPAP all night, it could be for other reasons: not sleeping enough hours, taking sedatives or having a low mood may also cause discomfort.<br><br>Answer: Yes<br>Great! The more hours you use CPAP, the better.
3) Are you less sleepy or tired during the day since using CPAP?	Answer: No<br>Do you use the CPAP enough hours? You must use it at least 4 hours, although the optimum is 6 hours. If you do not notice improvement despite wearing the CPAP all night, it could be for other reasons: not sleeping enough hours, taking sedatives or having a low mood may also cause discomfort.<br><br>Answer: Yes<br>Great! The more hours you use CPAP, the better.

4) Does CPAP cause congestion in your nose (such as a cold)?	Answer: Yes<br>You may need more time to adapt. Perform nasal washes with saline if you notice congestion and check the fit of the mask. If the problem persists, contact us and we will assess if you need the use of a chin guard to avoid leaks or a humidifier.<br><br>Answer: No<br>Great!
5) Do you notice that air comes out between the mask and your face?	Answer: Yes<br>It is very important that the size of the mask and its adjustment are adequate. Try to adjust the mask better and if the problem persists contact us to assess a change of mask.<br><br>Answer: No<br>Fantastic!
6) Do you notice that your mouth is dry with CPAP?	Answer: Yes<br>You may need more time to adapt. It may be because you open your mouth during sleep. If the problem persists, contact us and we will evaluate if you need the use of a chin holder, a full face mask or a humidifier.<br><br>Answer: No<br>Fantastic!
7) Do you perform the recommended physical exercise?	Answer: No<br>Exercise is essential to live better and avoid diseases, especially cardiovascular ones. In addition, it also improves apneas. See the sleep / health section: walking in a hurry half an hour each day may be enough!<br><br>Answer: Yes<br>Great! Exercise is essential to live better and avoid diseases, especially cardiovascular ones. In addition, it also improves apneas.
8) Do you comply with the recommended diet?	Answer: No<br>Obesity promotes many diseases such as diabetes, stroke and heart disease, among others. Consult dietary advice to follow a healthy diet and achieve a reduction in weight, which also helps improve apneas.<br><br>Answer: Yes<br>Fantastic! Losing weight improves health in general

	and also apneas. In addition, obesity promotes many diseases.
9) Do you comply with the recommendations that you have been given about sleep / health?	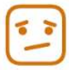 Answer: No<br>Sleeping is healthy.<br>It is indispensable for living better and avoiding illnesses and accidents. Consult the dream / health tips and try to follow them.<br>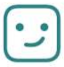 Answer: Yes<br>Fantastic! Sleeping well is a right, a duty and a pleasure. Keep it up!
10) Please introduce your body weight	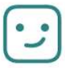 In a week we will ask for your weight again.
