# Supplementary material for: Telemedicine Strategy to Rescue CPAP Therapy in Sleep Apnea Patients with Low Treatment Adherence: A Pilot Study
Source: J Clin Med. 2021 Sep 13;10(18):4123. doi: 10.3390/jcm10184123 (PMC8470548; doi:10.3390/jcm10184123)
Supplement: Supplementary file 1 [file jcm-10-04123-s001.zip › Table S2 CPAP compliance (h per night).pdf]

Table S2. CPAP compliance (h/night)

(patients ordered according to PRE)

PRE	POST	1-MONTH
0	1.14	0.15
0	4.14	0.02
0	0.04	0.04
0.06	4.38	3.41
0.07	3.1	4.02
0.15	0.55	1.09
0.21	4.08	4.23
0.29	5.57	6.39
0.31	5.22	3.47
0.33	4	0.31
0.49	1.36	2.24
0.54	4.33	4.36
0.71	4.1	4.44
0.92	1.34	3.04
1.03	6.31	6.15
1.03	5.41	5.35
1.2	6.52	6.52
1.33	6.47	5.3
1.34	5.22	5.08
1.54	5.53	6.34
1.82	6.4	3.04
1.9	8.15	8.31
2.09	3.38	3.14
2.29	5.04	5.1
2.59	6.38	6.5
2.73	5.1	4.37
2.75	6.32	6.32
3	5.54	4.31
3.14	6.05	6.26
3.16	7.25	6.45
3.16	5.14	5.01
3.23	5.24	5.11
3.25	4.03	3.29
3.27	5.5	5.55
3.49	5.59	6.37
3.71	5.53	5.08
3.85	5.43	5.57
3.92	6.29	4.2
4.03	4.28	4.28
4.06	5.31	4.54
4.17	6.4	5.19

4.2	4.4	4.41
4.28	4.16	2
4.32	6.23	5.23
4.4	5.12	4.45
4.49	4.5	5.19
4.5	5.09	4.4
4.6	5.24	5.25
4.69	5.3	5.28
4.76	6.34	7.5
4.89	7.04	7.38
5.11	7.34	6.59
5.17	7.44	7.32
5.18	5.17	5.46
5.38	4.49	5.13
5.39	6.42	6.04
